# PolyMetformin combines carrier and anticancer activities for *in vivo* siRNA delivery

**DOI:** 10.1038/ncomms11822

**Published:** 2016-06-06

**Authors:** Yi Zhao, Wei Wang, Shutao Guo, Yuhua Wang, Lei Miao, Yang Xiong, Leaf Huang

**Affiliations:** 1 Division of Molecular Pharmaceutics, Eshelman School of Pharmacy, University of North Carolina at Chapel Hill, Chapel Hill, NC 27599, USA; 2 Center for Nanotechnology in Drug Delivery, Eshelman School of Pharmacy, University of North Carolina at Chapel Hill, Chapel Hill, North Carolina 27599, USA; 3 State Key Laboratory of Natural Medicines, Department of Pharmaceutics, China Pharmaceutical University, Nanjing 210009, China; 4 Department of Pharmaceutical Science, Zhejiang Chinese Medical University, Hangzhou 310053, Zhejiang, China

## Abstract

Metformin, a widely implemented anti-diabetic drug, exhibits potent anticancer efficacies. Herein a polymeric construction of Metformin, PolyMetformin (PolyMet) is successfully synthesized through conjugation of linear polyethylenimine (PEI) with dicyandiamide. The delocalization of cationic charges in the biguanide groups of PolyMet reduces the toxicity of PEI both *in vitro* and *in vivo*. Furthermore, the polycationic properties of PolyMet permits capture of siRNA into a core-membrane structured lipid-polycation-hyaluronic acid (LPH) nanoparticle for systemic gene delivery. Advances herein permit LPH-PolyMet nanoparticles to facilitate VEGF siRNA delivery for VEGF knockdown in a human lung cancer xenograft, leading to enhanced tumour suppressive efficacy. Even in the absence of RNAi, LPH-PolyMet nanoparticles act similarly to Metformin and induce antitumour efficacy through activation of the AMPK and inhibition of the mTOR. In essence, PolyMet successfully combines the intrinsic anticancer efficacy of Metformin with the capacity to carry siRNA to enhance the therapeutic activity of an anticancer gene therapy.

## Abstract (web summary)

The anti-diabetic drug Metformin also possesses anti-tumour activity. Here, the authors synthesize polymeric Metformin-based nanoparticles that still exert intrinsic biological activity through AMPK and mTOR regulation and can systematically deliver VEGF siRNA, significantly reducing lung cancer growth in mice.

## Graphical abstract

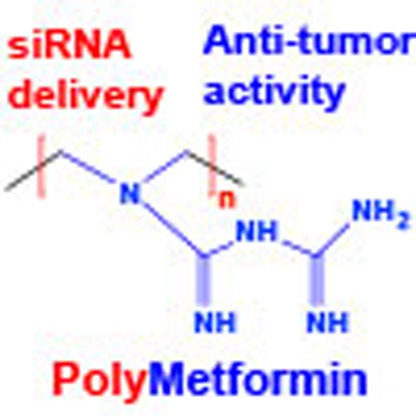

The discovery of RNA interference (RNAi) has since provided a conserved cellular pathway to modulate or inhibit endogenous gene expression through short interfering RNA (siRNA) therapeutics^1^,^2^. However, naked siRNA is susceptible to degradation by nucleases in blood serum and unable to cross the cell membrane due to its anionic charge^3^,^4^. Thus, systemic delivery of RNAi therapeutics to cancerous tissues remains a major challenge. To date, *in vivo* siRNA delivery has been achieved by vector-based delivery systems with cationic or ionizable characteristics, including polycation^5^,^6^, lipid^7^,^8^,^9^ and inorganic nanoparticles^10^,^11^, and RNA modifications/conjugations^12^,^13^. Among them, delivery using cationic polymers composed of different cationic compositions has become one of the most widely accepted strategies for delivery of siRNA^14^,^15^. Yet, the functional mechanisms of a polycation's molecular composition on siRNA delivery remain unclear^16^. Recently, materials have been developed incorporating guanidine groups to increase cellular uptake and transfection^17^,^18^. Herein a polycationic bi-guanidine composed of the anticancer therapeutic, Metformin (dimethyl-bi-guanide), has been developed for *in vivo* siRNA delivery. Metformin, one of the most effective drugs against diabetes^19^, is also known as a therapeutic agent against cancers including lung cancer^20^,^21^, pancreatic cancer^22^, breast cancer^23^, gastric cancer^24^,^25^ and so on. The anticancer efficacy of Metformin can be primarily attributed to the activation of AMP-activated protein kinase (AMPK)^26^,^27^ and inhibition of the mammalian target of rapamycin (mTOR)^28^,^29^. This cationic small molecular drug shows excellent tolerability and can be dosed at up to 2 g per day due to low toxicity. Moreover, the cationic biguanide composition of Metformin makes its polymeric form a desirable carrier for siRNA delivery. Therefore, it is expected that polymeric Metformin (PolyMet) would serves dual purposes as both a gene carrier and an antitumour therapeutic to achieve combinational therapeutic efficacies against cancer. Human non-small-cell lung cancer (NSCLC) is a well-known, aggressive and metastatic refractory tumour that responds to treatment by Metformin^30^,^31^. Thus, a NSCLC tumour cell H460 was used to evaluate the efficacy of siRNA delivery and antitumour abilities of PolyMet in this study.

Our data show a possibility to facilitate vascular endothelial growth factor (VEGF) siRNA *in vivo* delivery by PolyMet nanoparticles and enhanced tumour growth. In the absence of RNAi, LPH-PolyMet nanoparticles, like metformin, activated the AMPK, inhibited the mTOR pathway and induced tumour autophagy and apoptosis. Thus, PolyMet successfully combines the intrinsic anticancer efficacy of Metformin with the capacity to carry siRNA to enhance the therapeutic activity of an anticancer gene therapy.

## Results

### Synthesis and characterization of PolyMet polymer

**Figure 1:**
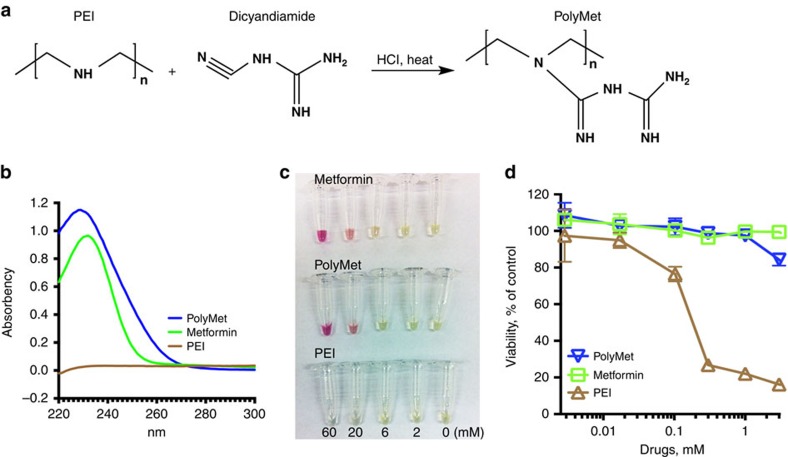
Synthesis and characterization of PolyMet. (**a**) Synthesis scheme of PolyMet polymer. (**b**) Ultraviolet spectra of Metformin, PEI and PolyMet in the range of 220–300 nm. (**c**) Colour test of Metformin, PEI and PolyMet. Test reagents were prepared by mixing equal volumes of 10% w/v sodium nitroprusside with 10% w/v potassium hexacyanoferrate (III) and 10% sodium hydroxide. Equal amounts of Metformin, PEI unimer or PolyMet unimer in aqueous solution were mixed with 100 μl of the test reagent. The image was taken 30 min after mixing. (**d**) Cytotoxicity of Metformin, PEI and PolyMet. H460 cell availability was measured using a 3-(4,5-dimethylthiazol-2-yl)-2,5-diphenyltetrazolium bromide (MTT) assay after 24 h of exposure to Metformin, PolyMet and PEI solutions. Data are mean±s.d. (*n*=8). Data are representative of **b** and **c** or combined (**d**) from three independent experiments.

Inspired by the fact that Metformin can be synthesized through a one-step reaction of dimethylamine hydrochloride and 2-cyanoguanidine (dicyandiamide), with heating (Supplementary Fig. 1), the Metformin polymer was designed using a similar method. To yield PolyMet, linear polyethylenimine (PEI) and dicyandiamide were reacted under heat in acidic conditions (Fig. 1a). Linear PEI hydrochloride (0.2 g) and dicyandiamide (2 g) were mixed in 10 ml 2 M HCl solution. The reaction mixture was reacted at 100 °C for 24 h, purified through an ultrafiltration tube to remove excess dicyandiamide, washed with deionized water for two times and lyophilized. The formation of PolyMet was verified by using proton nuclear magnetic resonance (^1^H-NMR) (Supplementary Fig. 2A) and matrix assisted laser desorption/ionization time-of-flight (MALDI-TOF) (Supplementary Fig. 2B,C) analyses. ^1^H-NMR (400 MHz, d6-dimethylsulfoxide) spectrum exhibits all characteristic proton resonance peaks corresponding to the present PolyMet molecules. The appearance of the proton resonance at *δ* 2.86–3.75 p.p.m. in the product along with the characteristic guanidium proton at *δ* 5.80–6.40 p.p.m. confirmed the formation of biguanide polymer. The spectrum also indicated near 95% substitution ratio of PolyMet by comparing the area ratios of PEI (2.53–2.70 p.p.m.) and PolyMet (2.86–3.75 p.p.m.). MALDI-TOF of the PolyMet and free PEI was performed to determine the synthesis of PolyMet (Supplementary Fig. 2B,C). The distribution centre for PEI (ca. 570 Da) was completely red-shifted compared with PolyMet (ca. 1600 Da), which is consistent with the ^1^H-NMR result, indicating successful conjugation of PEI with 2-cyanoguanidine (Supplementary Fig. 2B). The average molecular weight of PEI shown by MALDI-TOF analysis is smaller than the molecular weight we used for synthesis (ca. molecular weight is 4,300 Da), which might be due to the existence of numerous differently charged species of PEI, since MALDI-TOF only detects singly charged species^32^. Moreover, the extended MALDI-TOF mass spectra (Supplementary Fig. 2C) show several series of oligomer ions 43 and 129 *m*/*z* apart from each other, in agreement with the PEI (43 *m*/*z* for C_2_H_5_N unimer) and PolyMet (129 *m*/*z* for C_4_H_11_N_5_ unimer) repeat units, respectively. As described in authoritative literatures^33^,^34^, Metformin can be characterized by (1) using ultraviolet absorption at the wavelength of maximum absorbance at about 233 nm or (2) exhibiting a red colour in the solution of nitroprusside/potassium hexacyanoferrate(III)/sodium hydroxide. Both PolyMet and Metformin exhibited a maximum absorbance around 233 nm (Fig. 1b) and showed red colour in the colour test in a dose-dependent manner (Fig. 1c). These results suggest that after the reaction with dicyandiamide, the secondary amines in PEI had been completely replaced with biguanides.

Notably, PolyMet showed a significantly lower cytotoxicity (Fig. 1d) and threefold higher maximum tolerable dose compared with PEI (Supplementary Table 1), indicating PolyMet containing guanidine is more tolerable than its secondary amine-containing counterpart. But the lower maximum tolerable dose of PolyMet than Metformin (Supplementary Table 1) is perhaps attributed to the interaction of the cationic polymer with the serum proteins in the blood. In summary, PolyMet as a cationic polymer may be used to condense and deliver negatively charged siRNA with similar pharmacological properties and activity to Metformin due to the biguanide structure.

### Preparation and characterization of LPH nanoparticles

**Figure 2:**
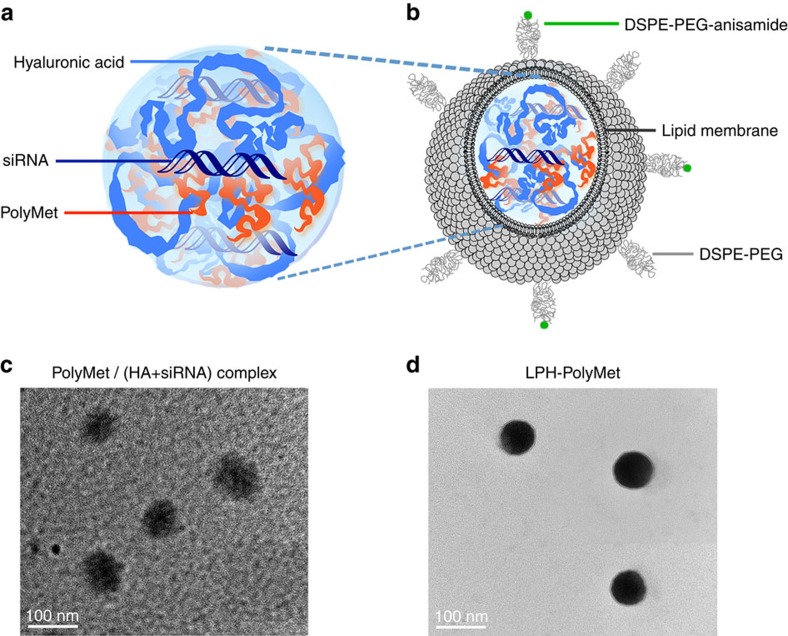
Schematic illustration and representative TEM images of PolyMet nanoparticles. Anionic HA+siRNA mixture was condensed by cationic PolyMet into a negatively charged PolyMet/(HA+siRNA) complex (**a**,**c**). DOTAP/cholesterol cationic liposomes were added to the complex to form lipid coating, then DSPE-PEG and DSPE-PEG-anisamide were used to liposome by the post-insertion method to form LPH-PolyMet final nanoparticles (**b**,**d**).

It is well documented that siRNA forms a loose and unstable complex when mixed with only polycationic polymers due to its low molecular weight and low charge density^35^,^36^. Therefore, liposomes were used to coat complexes to protect siRNA from dissociation or clearance before it reaches the tumour in our study. First of all, hyaluronic acid (HA), a polyanionic and multivalently charged polysaccharide, was used to facilitate siRNA delivery and enhance the particle condensation. Then cationic 1,2-dioleoyl-3-trimethylammonium-propane chloride salt (DOTAP) liposome was applied to coat complex to prove the stability of complex. These core/shell-type nanoparticles, referred to the lipid-polycation-hyaluronic acid (LPH) nanoparticle were established for systemic delivery of siRNAs to the tumour site^37^,^38^. Specifically, HA was mixed with siRNA at 1:1 mole ratio, and modulated the N/P ratios of PolyMet/(HA+siRNA) complex. Aggregates were observed at an N/P ratio around 0.9, where a neutral complex was formed. In this case, 0.6 was chosen as the optimal ratio, as the complex remained negatively charged (∼ −20 mV) with a relatively small size (∼100 nm). At this ratio the PEI/(HA+siRNA) complex had a similar charge and size with PolyMet/(HA+siRNA) complex (Supplementary Fig. 3). Next, DOTAP/cholesterol (1:1 mol/mol) cationic liposomes were added to the complex to form the lipid coating through charge–charge interactions. Studies have shown that the NSCLC cell H460 overexpresses sigma receptors^17^,^39^,^40^, thus a targeting ligand for the sigma receptors, DSPE-PEG-anisamide, was used to deliver nanoparticles to the tumour by the post-insertion method to form the outer leaflet of the liposome membrane. The size, distribution, morphology and the characteristics of the nanoparticles are summarized in Fig. 2 and Supplementary Table 2. The lipid coating reduced the complex's size due to electrostatic interactions. The final nanoparticles were 70–80 nm with a positive charge of about 20 mV and 4.38% loading efficiency (Supplementary Table 2).

### LPH-PolyMet combines siRNA carrier and anticancer activities

**Figure 3:**
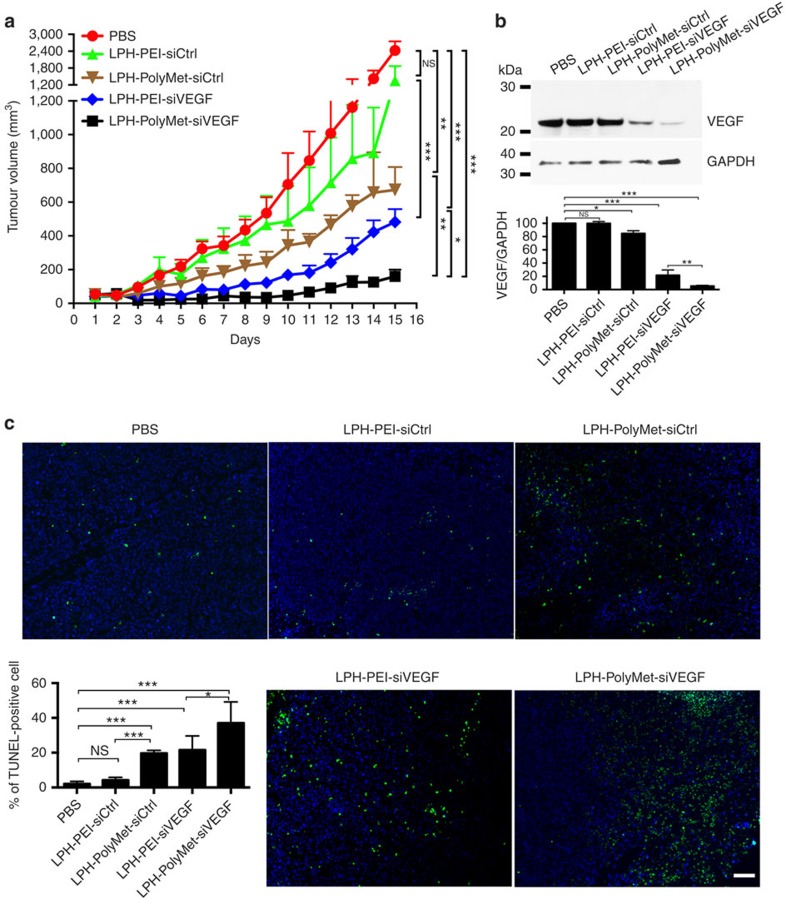
LPH nanoparticles composed of PolyMet can systemically deliver VEGF siRNA to the tumour site and inhibit tumour growth. (**a**) H460 tumour-bearing mice were injected i.v. every other day and tumour volumes were measured every day. (**b**) H460 tumour VEGF protein levels after two injections were measured by western blot analysis. Bar chart in **b** represent quantitative analysis of relative normalized VEGF band intensity (Image J). (**c**) TUNEL staining (green) in H460 tumour cells after treatment with siRNA in different formulations *in vivo*. Nucleic acid was stained with 4,6-diamidino-2-phenylindole (blue). Bar chart in **c** is a quantitative analysis of % of TUNEL-positive cells. Five randomly selected microscopic fields were quantitatively analysed on Image J. Data are mean±s.e.m. (*n*=5 per group) analysed by two-way analysis of variance with Tukey's correction. Data are representative of **b** and **c** or combined from (**a**) three independent experiments. NS, not significant; *P<0.05, **P<0.01, ***P<0.005. Scale bar, 200 μm.

Previous studies have shown that VEGF siRNA suppresses angiogenesis and may be a potential therapeutic agent for NSCLC^41^. Human NSCLC H460 tumour-bearing mice received intravenous (i.v.) injections of different formulations with VEGF siRNA or control siRNA every other day. On successive i.v. injections into tumour-bearing mice, the greatest enhancement of antitumour efficacy was observed when LPH-PolyMet-siVEGF was administered (Fig. 3a).

Notably, the LPH-PolyMet-siCtrl group that contained control siRNA also showed a partial tumour suppression activity, indicating that poly-biguanide structure of PolyMet has the antitumour activity. Meanwhile, minimal suppression of tumour growth was found in the LPH-PEI-siCtrl group. One day after the second injection, mice were killed and tumour lysates were prepared for western blot. As shown in Fig. 3b, LPH-PolyMet-siVEGF group showed robust knockdown of VEGF with about 95% of silencing compared with the phosphate-buffered saline (PBS) group. LPH-PEI-siVEGF had only a partial effect on the VEGF expression level with about 70% of knockdown in comparison with control group. LPH-PolyMet-siCtrl showed a slight knockdown effect. LPH-PEI-siCtrl had no measurable effect on the protein expression level of VEGF.

Terminal deoxynucleotidyl transferase dUTP nick end labelling (TUNEL) assay further confirmed the induction of apoptotic cells in tumours (Fig. 3c). The number of TUNEL-positive apoptotic cells after LPH-PolyMet-siVEGF treatment was about 40%, which was the highest among all groups. This indicates that elevated apoptosis occurred after downregulation of VEGF expression, which is consistent with studies reported elsewhere^42^. Treatment with LPH-PEI-siVEGF could only induced apoptosis in about 20% of cells. Importantly, we also observed that LPH-PolyMet-siCtrl exhibited significant higher TUNEL-positive population compared with PBS or LPH-PEI-siCtrl, indicating the apoptotic capability of PolyMet gene carrier itself.

PolyMet LPH was then investigated to determine its ability to carry other therapeutic siRNA. As an anti-apoptotic protein BCL-2 promotes cell survival, and inhibition of BCL-2 can suppress tumour growth^43^,^44^. Herein siRNA used against BCL2 inhibited tumour growth, especially in the LPH-PolyMet-siBCL2 treatment group, which provided persistent suppression of tumour growth (Supplementary Fig. 4A). The level of BCL-2 after treatment was detected by western blot analysis, LPH-PolyMet-siBCL2 induced a significant downregulation of BCL-2 level in comparison with all other groups (Supplementary Fig. 4B) and the TUNEL assay further confirmed the induction of apoptotic cells in tumours. The number of TUNEL-positive apoptotic cells after LPH-PolyMet-siBCL2 treatment was about 70%, which was significantly higher than all other groups (Supplementary Fig. 4C). This indicates that elevated apoptosis occurred after downregulation of BCL2 anti-apoptotic protein expression in the LPH-PolyMet-siBCL2 group.

PolyMet was then tested for gene delivery and tumour inhibition in a melanoma model. As shown in Supplementary Fig. 5, the most effective treatment, LPH-PolyMet-siVEGF resulted in a knockdown of VEGF level by ∼70% and a suppression of tumour growth by about 80% in comparison with the PBS group in a 1205Lu melanoma model. Meanwhile, LPH-PEI-siVEGF also suppressed tumour growth effectively, but not as persistently as LPH-PolyMet-siVEGF.

Moreover, the antitumour activity of PolyMet was compared with other two commonly used polycations, poly-L-lysine (PLL) and protamine. PLL and Protamine were formulated in the LPH systems containing the same amount of VEGF siRNA as PolyMet. The antitumour efficacy and VEGF knockdown were presented in Supplementary Fig. 6. All LPH formulations had significantly suppressed tumour growth in comparison with PBS. However, there was no significant difference among PEI, PLL and protamine. The greatest enhancement of antitumour effect was observed with PolyMet. The expression of VEGF was also evaluated after day 16, as shown in Supplementary Fig. 6B. The VEGF level in the tumour was significantly lower than other groups, as well as the PBS control. Thus, PolyMet showed the greatest antitumour activity among all four polycations tested.

### Mechanisms of PolyMet on the inhibition of tumour growth

**Figure 4:**
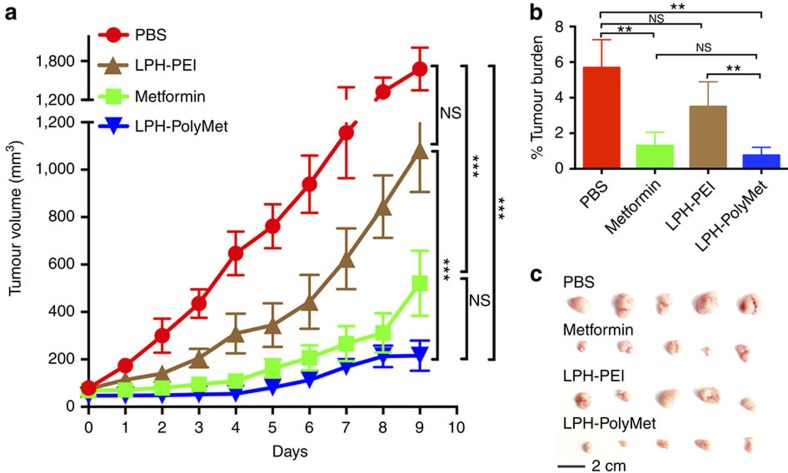
Metformin and PolyMet inhibit H460 tumour growth. PBS, Metformin, LPH-PEI and LPH-PolyMet were administered i.v. every other day, and mice were killed 24 h after the final injection. (**a**) Tumour volumes were measured every day. (**b**) Tumour weights were measured on day after final injection and compared with body weights to determine the percentage of tumour burden. (**c**) Visual observations of the H460 tumour sizes in each treatment group at the end time point; Data are mean±s.e.m. (*n*=5 per group) analysed by two-way analysis of variance with Tukey's correction. Data are combined from **a** and **b** or representative of (**c**) three independent experiments. NS, not significant; *P<0.05, **P<0.01, ***P<0.005.

Both clinical and preclinical studies have shown that Metformin improved survival and/or antitumour efficacy^25^,^45^. Results shown in Fig. 3 and Supplementary Figs 4, 5 and 6B indicate the tumour growth inhibition of LPH-PolyMet-siCtrl. It was thus hypothesized that in the absence of RNAi, PolyMet, like its unimer Metformin, may be responsible for some part of anticancer activity. The effect of LPH-PolyMet and different nanoparticles on an H460 xenograft was studied. As shown in Fig. 4, treatment with LPH-PolyMet led to significant inhibition of cancer growth in comparison with PBS. A noticeable difference in tumour growth inhibition was observed between LPH-PEI and LPH-PolyMet (Fig. 4a), suggesting that PolyMet played an important role in enhancing antitumour activity. At the end of the experiment, the tumours of the animals treated with LPH-PolyMet were <1% of the total body weight, which was significantly smaller than the LPH-PEI (4%) and PBS (6%) groups (Fig. 4b). Moreover, no toxicity was detected in blood haematology serum chemistry or the histology of the major tissues (Supplementary Fig. 7 and Supplementary Table 3).

Furthermore, we compared the effect of LPH-PolyMet siRNA nanoparticles with Metformin in an H460 xenograft (Supplementary Fig. 8). Free Metformin (1.25 mg kg^−1^) equivalent to the unimer dose of PolyMet (1.25 mg kg^−1^) that was required to deliver 0.625 mg kg^−1^ of VEGF siRNA was used in the study. It was confirmed that there is no significant difference between the PolyMet carrier (LPH-PolyMet-siCtrl) and Metformin, indicating that biguanide in the PolyMet copolymers are associated with the anticancer activities of Metformin. Both of the groups exhibit significantly higher antitumour growth activity compared with LPH-PEI-siCtrl or PBS while LPH-PolyMet-siVEGF has the best antitumour efficacy. In this case tumour growth was suppressed seven- to eightfold compared with the PBS group, and about twofold compared with the PolyMet carrier or the Metformin group, which suggests again that PolyMet successfully combine the intrinsic anticancer efficacy of Metformin with the tumour suppressive effects of VEGF siRNA to enhance the therapeutic activity of an anticancer gene therapy.

**Figure 5:**
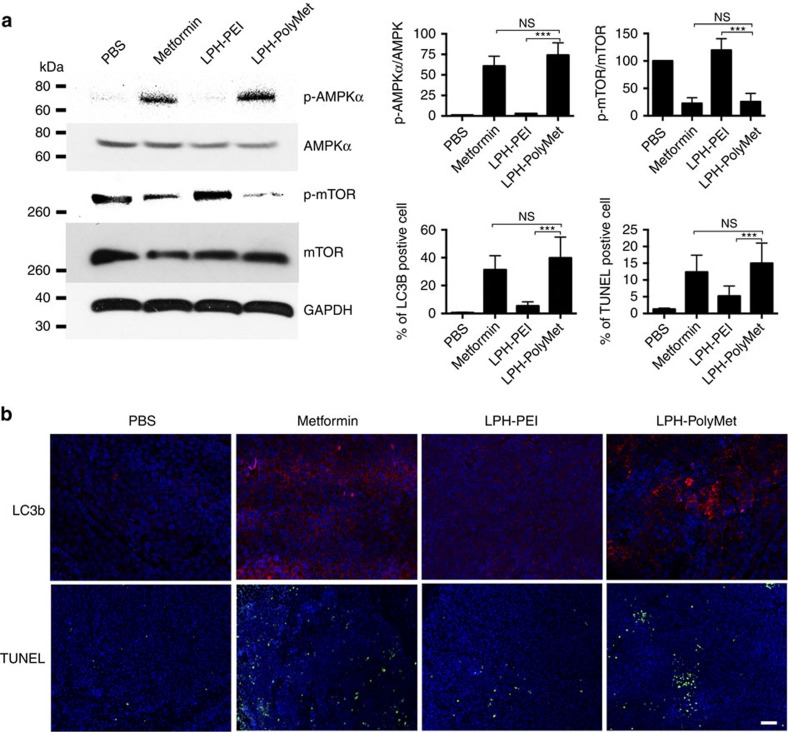
Metformin and PolyMet inhibit tumour growth by activating the AMPK and inhibiting the mTOR pathways, inducing autophagy and apoptosis mechanisms. Mice bearing H460 tumours were given i.v. injections every other day and tumour proteins was prepared 24 h after the second injection for analysis. Bar charts in (**a**) represent quantitative analysis of normalized p-AMPK and p-mTOR band intensity using Image J. Data are shown as mean±s.d. (*n*=3). Cells under autophagy were stained by LC3b antibody (red) and apoptosis of cells was indicated by TUNEL assay (green). Nuclei were stained blue. Bar charts in (**b**) are quantitative analysis of percentage of LC3b-positive cells and the percentage of TUNEL-positive cells, respectively. Five randomly selected microscopic fields were analysed using Image J. Data are analyzed by two-way analysis of variance (ANOVA) with Tukey's correction. Data are shown as mean±s.d. (*n*=5) and represent three independent experiments; NS, not significant; *P<0.05, **P<0.01, ***P<0.005. Scale bar, 200 μm.

It has been reported that Metformin inhibits cancer cells by activating the AMPK and inhibiting the mTOR pathways^28^,^46^. We checked the activity of PolyMet in these pathways and compared it with that of Metformin. AMPK acts as a metabolic tumour suppressor that governs glucose and lipid metabolism^47^. In many cases, a low level of phosphorylation of AMPK is correlated with poor prognosis after cancer treatment^48^. We observed that following treatment with Metformin or LPH-PolyMet, phosphorylation levels of AMPKα in the H460 tumour were significantly enhanced compared with the PBS group (Fig. 5a). The activation of AMPKα did not occur in the LPH-PEI group, suggesting PolyMet is the key factor in the nanoparticle that can activate AMPKα pathway. mTOR is a downstream effector of AMPK^49^. AMPK activation inhibits mTOR and its downstream effector kinases^50^. Phosphorylation of mTOR plays a pivotal role in the proliferation and survival of cancer cells^51^. Therefore, we then evaluated the effects of treatments on the activity of mTOR (Fig. 5a). Metformin and LPH-PolyMet treatments led to a significant inhibition of mTOR activity indicated by stronger reduction in p-mTOR/mTOR levels compared with the PBS group. The activity was not shared by LPH-PEI.

Autophagy is recognized as a potentially toxic mechanism for Metformin that results in the inhibition of cancer growth^52^,^53^. We evaluated whether autophagy can also be observed after treatment of LPH-PolyMet. Microtubule-associated protein light chain 3 b (LC3b) is a specific marker for autophagy initiation^54^. As shown in Fig. 5b, Metformin- and LPH-PolyMet-treated tumours showed a higher LC3b-associated red fluorescence than other groups, indicating that both Metformin and LPH-PolyMet could induce autophagy in the H460 lung cancer xenograft model.

Finally, the mechanism of the antitumour efficacy after treatment was determined by the TUNEL assay, a common method to test for apoptosis (Fig. 5b). The per cent of apoptotic cells after Metformin or LPH-PolyMet treatments was 12.4% and 15%, respectively, while no significant apoptosis induction was observed in other groups. This suggests that Metformin or polymer form can induce cell apoptosis and play a critical role in regulating the cancer cell survival.

## Discussion

Nanotechnology has been extensively exploited to prolong the circulation time and to enhance the therapeutic activity for a variety of drugs. After systemic injection the nanoparticles drain through the leaky tumour blood vessels and are retained at the tumour site, resulting in increased tumour accumulation of the nanoparticle-formulated drug compared with the free drug. This well-documented phenomenon is referred to as the enhanced permeability and retention effect^55^. Thus, nanoparticles offer a number of advantages over free drugs, which make them an ideal delivery vehicle for Metformin. However, due to its high hydrophilicity, Metformin cannot be effectively loaded into established nanocarriers, which explains why only few Metformin nanoparticles have been reported^56^,^57^,^58^. Recently, Yang *et al*.^56^ reported the co-encapsulation of epirubicin and Metformin into liposomes through remote loading. This nanoparticle successfully inhibited the growth of CD133+ cancer stem-like cells. Jose *et al*.^57^ developed Metformin-loaded bovine serum albumin nanoparticles (MET-BSA) by the coacervation method and observed that MET-BSA nanoparticle showed higher efficacy than Metformin alone or the BSA carrier itself. However, PolyMet, which combines both gene delivery and anticancer capabilities, represents a novel approach.

Guanidination of a polymer enhances the activity for gene delivery and has considerably less toxicity compared with primary amines of the polymer^59^,^60^. The guanidine has been found to readily pass through the non-polar membrane of a cell and even across tissue barriers by possibly forming a bidentate hydrogen bond with anionic cell surface phosphate, carboxylates and/or sulfates on the cell surface^61^,^62^. Materials have been previously developed incorporating guanidine groups to increase cellular uptake and transfection^60^,^63^,^64^,^65^. For example, Lee *et al*.^60^ used pyrazole-1-carboxamidine to react with branched PEI, and observed enhancement of transfection efficiency and decreased cytotoxicity of PEI by guanidylation. Wender *et al*. developed non-natural guanidinium-rich molecular transporters to enhance cellular uptake^61^,^66^, through tunable polyguanidino dendrimers, and observed higher cellular uptake^65^. Guanidinium-rich molecular transporters can penetrate cells and overcome efflux-mediated multidrug resistance^67^. Although these previously reported polyguanidines are established gene carriers, the carriers themselves fail to exhibit anticancer activity without their therapeutic agent. Thus, PolyMet serves as both a gene carrier and anticancer therapeutic. However, findings presented herein have not been investigated with PolyMet against a p53-deficient tumour, which remains to be further explored. The focus of this study is to show the carrier and anticancer activities of PolyMet. The dose of PolyMet used in the study was dictated by the dose of siRNA and was only a fraction of the effective dose of metformin. Further study with increased PolyMet dose will bring the new polymer a step closer to the clinical translation.

## Methods

### Materials

DOTAP and 1,2-distearoryl-sn-glycero-3-phosphoethanolamine-*N*-[methoxy(polyethyleneglycol-2000) ammonium salt (DSPE-PEG_2000_) were purchased from Avanti Polar Lipids, Inc. (Alabaster, AL). DSPE-PEG-anisamide was synthesized in our lab as described previously^68^. DeadEnd Fluorometric TUNEL assay kits and Luciferase Assay System assay substrates were obtained from Promega (Madison, WI). Linear PEI hydrochloride with average molecular weight 8,000, dicyandiamide, HA and other chemicals were obtained from Sigma-Aldrich (St. Louis, MO). Rabbit monoclonal antibodies Phospho-AMPKα(Thr172) (40H9) (catalogue # 2535), AMPKα (D5A2) (catalogue # 5831), Phospho-mTOR (Ser2448) (D9C2) XP (catalogue # 5536), mTOR (7C10) (catalogue # 2983), LC3b (D11) XP (catalogue # 3868), GAPDH (14C10) (catalogue # 2118) and Anti-rabbit IgG, horseradish peroxidase-linked Antibody (catalogue # 7074) were purchased from Cell Signaling Technology (Beverly, MA). BCL2 siRNA (target sequence: 5′-AACAUCGCCCUGUGGAUGACU-3′), VEGF siRNA (target sequence: 5′-ACCUCACCAAGGCCAGCAC-3′) and control siRNA (target sequence: 5′-AAUUCUCCGAACGUGUCACGU-3′) were synthesized by Sigma-Aldrich.

### Cell culture

H460 human NSCLC cells and 1205Lu melanoma cancer cells were originally obtained from American Type Culture Collection and were cultured in DMEM medium (Invitrogen, Carlsbad, CA) supplemented with 10% fetal bovine serum (Life Technologies, Carlsbad, CA), 100 U ml^−1^ penicillin and 100 μg ml^−1^ streptomycin (Invitrogen). Cells were cultured in a humidified incubator at 37 °C and 5% CO_2_.

### Experimental animals

Female nude female mice and female CD1 mice that were 6–8 weeks old were used in the studies. Female nude mice were purchased from National Cancer Institute (Bethesda, MD) and bred by the Division of Laboratory Animal Medicine at University of North Carolina at Chapel Hill. CD1 mice were purchased from Charles River Laboratories (Morrisville, NC). To establish the xenograft models, 5 × 10^6^ cells in 100 μl of PBS were injected subcutaneously into the right flank of the mice. All work performed on animals was approved by the Institutional Animal Care and Use Committee at the University of North Carolina at Chapel Hill.

### Preparation of PolyMet

To prepare PolyMet, 0.2 g of linear PEI and 2 g of dicyandiamide were mixed in 10 ml 2 M HCl solution. The compounds reacted at 100 °C in the oil bath for 24 h. PolyMet was then purified through an ultrafiltration tube, washed with deionized (DI) water for two times and lyophilized. The working solution of PolyMet was diluted in DI water and kept as 1 mg ml^−1^, 1 ml aliquots for experiment. To verify the formation of PolyMet, NMR and MALDI-TOF analyses were performed (Supplementary Methods).

### Optimization of the PolyMet-HA or PEI-HA complexes

A volume of 200 μl of PEI solution (containing 5, 10, 15 or 20 μg PEI in DI water) or 200 μl of PolyMet solution (containing 50, 60, 70 or 80 μg PolyMet in DI water) was mixed with 200 μl of HA (containing 50 μg HA, in DI water) in a 1.5-ml tube. The complexes were mixed by pipetting up and down for 10 times and allowed to stand at room temperature for 10 min. The optimal ratio of the complex was determined by the results from particle size and zeta potential determined by dynamic light scattering using a Malvern ZetaSizer Nano series (Westborough, MA).

### Preparation of LPH nanoparticles

DOTAP/cholesterol liposomes were prepared as follows: DOTAP (20 mM, 1 ml) and cholesterol (20 mM, 1 ml) were dissolved (1:1 mol/mol) in chloroform and the solvent was removed under reduced pressure. The lipid film was then hydrated overnight with 2 ml distilled water to form cationic liposomes (10 mM), which were sequentially extruded through polycarbonate membranes (200 nm × 20 times, 100 nm × 20 times and 50 nm × 20 times) (Millipore, Billerica, MA). LPH cores were prepared by mixing 100 μl of 5% glucose solution A (containing the 12.5 μg of siRNA and 12.5 μg of HA) with 100 μl of 5% glucose solution B (containing 5 μg of PEI, or 25 μg of PolyMet, or 6 μg of PLL, or 13 μg of Protamine) and incubated at room temperature for 10 min. The LPH cores were then mixed with 30 μl of cationic DOTAP/cholesterol liposomes and incubated at room temperature for 10 min to ensure proper lipid coating. The lipid-coated nanoparticles were PEGylated using a post-insertional approach by adding 10 μl DSPE-PEG (10 mg ml^−1^) and 10 μl DSPE-PEG-anisamide (10 mg ml^−1^) and incubating the nanoparticles at 50 °C for 15 min.

### Characterization of LPH NPs

The size and surface charge of LPH cores and final particles were determined by using a Malvern ZS90 Zetasizer (Worcestershire, UK). Transmission electron microscope images of LPH were acquired through the use of JEOL 100CX II transmission electron microscope (Tokyo, Japan). Briefly, freshly prepared LPH nanoparticles (5 μl) were carefully dropped onto a 300-mesh carbon-coated copper grid (Ted Pella, Inc., Redding, CA) and allowed to stand at room temperature for 5 min. Grids were then stained with 1% uranyl acetate (5 μl) and allowed to incubate briefly (10 s) and quickly dry. All images were acquired at an accelerating voltage of 100 kV.

### Loading capacity of LPH nanoparticles

After final LPH-PolyMet-siRNA formulation, the free siRNA were separated by centrifugation for 20 min at 18,000*g* and 4 °C. The siRNA concentration in the supernatant was measured by the Quant-iT RiboGreen RNA reagent according to the manufacturer's instructions, using a fluorescence spectrometer (Perkin-Elmer, Waltham, MA) at 485/520 nm excitation/emission. Each sample was assayed in triplicate.


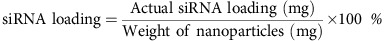


### Animal tumour model and antitumour activity

H460 human lung cancer cells (5.0 × 10^6^) were subcutaneously injected into the right flanks of female athymic nu/nu mice. Drugs were administrated i.v. into mice when the tumours reached about 50–80 mm^3^ in size (5–7 days after transplantation). LPH equivalent to 0.625 mg kg^−1^ of VEGF siRNA or control siRNA, prepared as described above, were i.v. injected every other day. Free Metformin (1.25 mg kg^−1^) equivalent to the unimer dose of PolyMet (1.25 mg kg^−1^) that was required to deliver 0.625 mg kg^−1^ of siRNA was used in the study. Animal weight and tumour volumes were measured every other day. The tumour length (*L*) and width (*W*) were used to calculate volume (*V*) by the following equation: *V*=1/2 × *L* × *W*^2^. Serum biochemical value analysis and haematology assay were provided in Supplementary Methods.

### Western blot analysis

Western blotting was used to evaluate the protein expression of tumour cells after systemic administration. Tumour-bearing mice were injected i.v. every other day and were killed 24 h after the second injection. A unit of 50 mg of tumour was collected randomly via biopsy and homogenized with a Tissue Master-125 homogenizer (OMNI International) in 500 μl cold radioimmunoprecipitation assay buffer (Sigma-Aldrich) supplemented with 1% protease inhibitor cocktail (Sigma-Aldrich) for 30 min. Total homogenate was then spun down at 13,000*g* with a tabletop centrifuge for 10 min at 4 °C. Supernatant was collected and protein sample was quantified using a bicinchoninic acid assay kit in accordance with the manufacturer's instruction (Pierce Biotech). Samples were diluted in 4 × sample buffer and 10 × sample-reducing reagent (Thermo Fisher). A unit of 40 mg protein from each group was separated by 4–12% SDS–PAGE electrophoresis (Invitrogen) and transferred to a polyvinylidene difluoride membrane (Bio-Rad). Membranes were blocked with 5% non-fat dry milk (Bio-Rad) for 1 h at room temperature and then incubated with primary antibodies (1:1,000 dilution; Cell Signal) overnight at 4 °C. The membranes were washed three times with PBS-Tween, followed by incubation with horseradish peroxidase-conjugated secondary antibody (1:4,000 dilution; Cell Signal) for 1 h at room temperature. Finally, the membranes were washed three times with PBS-Tween and developed using Pierce ECL Western Blotting substrate (Thermo Scientific). The expression level of targeted protein was quantified by ImageJ software (NIH). GAPDH was detected as loading control. Images have been cropped for presentation but are otherwise unmodified. Full-size images are presented in Supplementary Figs 9 and 10.

### Immunostaining

The TUNEL assay was used to detect apoptotic tumour cells after systemic administration. Briefly, tumours were collected and fixed in freshly prepared 4% paraformaldehyde or 10% formalin for 1 day. Tumour samples were paraffin-embedded and sectioned by the UNC Animal Histopathology Core. The slides were then deparaffinized in xylene and rehydrated through a gradient alcohol series. The TUNEL staining was performed in accordance with the manufacturer's protocol (Promega). Nuclei of tumour cells were stained with DAPI (Vector Laboratories). Images of were taken using a fluorescence microscope (Nikon). The percentage of apoptotic cells was calculated by dividing the number of apoptotic cells from the total number of cells in each microscopic field. Representative microscopic fields (*n*=5) were randomly selected in each treatment group for this analysis.

### Statistics analysis

All statistical analyses were carried out using GraphPad Prism Software (Version 5.0, GrapPad Software, San Diego, CA).

## Additional information

**How to cite this article:** Zhao, Y. *et al*. PolyMetformin combines carrier and anticancer activities for *in vivo* siRNA delivery. *Nat. Commun.* 7:11822 doi: 10.1038/ncomms11822 (2016).

## Supplementary Material

Supplementary Information Supplementary Figures 1-10, Supplementary Tables 1-3 and Supplementary Methods
