# Supplementary material for: PolyMetformin combines carrier and anticancer activities for in vivo siRNA delivery
Source: Nat Commun. 2016 Jun 6;7:11822. doi: 10.1038/ncomms11822 (PMC4897747; doi:10.1038/ncomms11822)
Supplement: Supplementary Information — Supplementary Figures 1-10, Supplementary Tables 1-3 and Supplementary Methods [file ncomms11822-s1.pdf]

Supplementary Figures

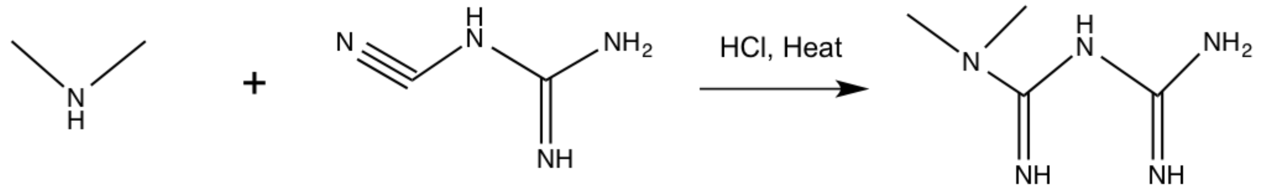

Supplementary Figure 1. Synthesis routine of Metformin.

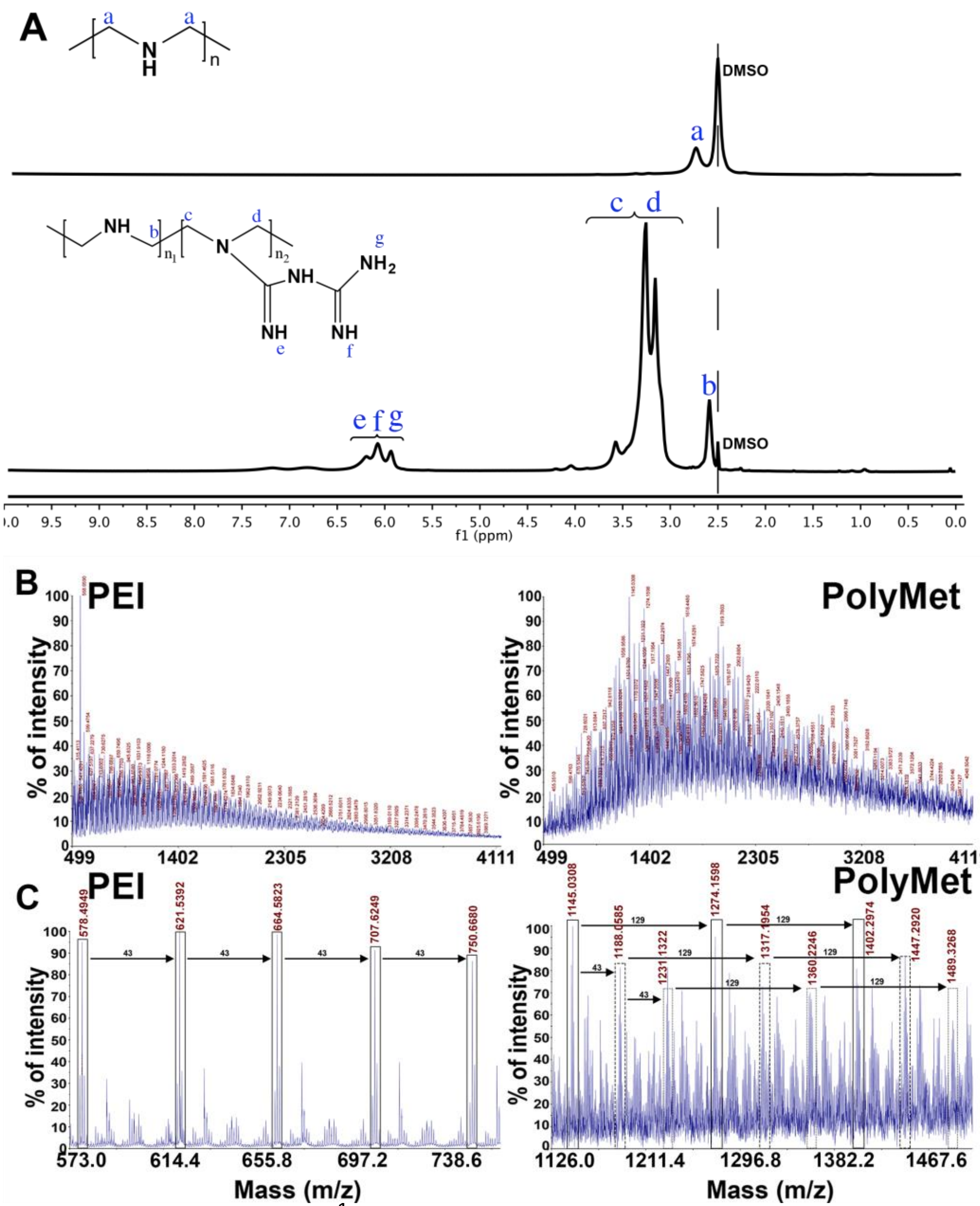

Supplementary Figure 2. (A) ^{1}H-NMR spectrums of PEI (top), and partially reacted product PEI-PolyMet (bottom) in d6-DMSO. The picture in each spectrum is the partial structure of the polymer with alphabetic labels representing major protons in the polymer chain, n=n1+n2. After the reaction, the polymer was isolated by filtration, then adjusted to pH 12 with 10 M NaOH, dialyzed against DI-water for 2 days and subsequently lyophilized. The appearance of the proton resonance at δ 2.86 to 3.75 ppm in the product along with the characteristic biguanide protons at δ 5.80 to 6.40 ppm confirmed the formation of biguanide polymer. The spectrum also indicated near 95% substitution ratio of PolyMet by comparing the area ratios of PEI (2.53 to 2.70 ppm) and PolyMet (2.86 to 3.75 ppm). **(**B and C) MALDI-TOF mass spectra of PEI and PolyMet. The MALDI-TOF samples were prepared in 50% acetonitrile solution, using a α-Cyano-4-hydroxycinnamic acid matrix.

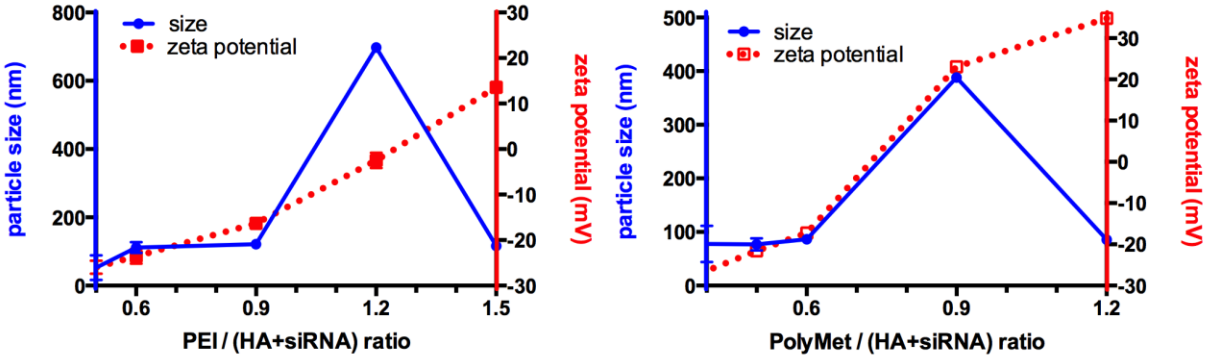

Supplementary Figure 3. Formulation optimization of the complexes. Effect of N/P ratio of PEI/(HA+siRNA) (A) and PolyMet/(HA+siRNA) (B) on particle size (blue) and zeta potential (red) of complexes.

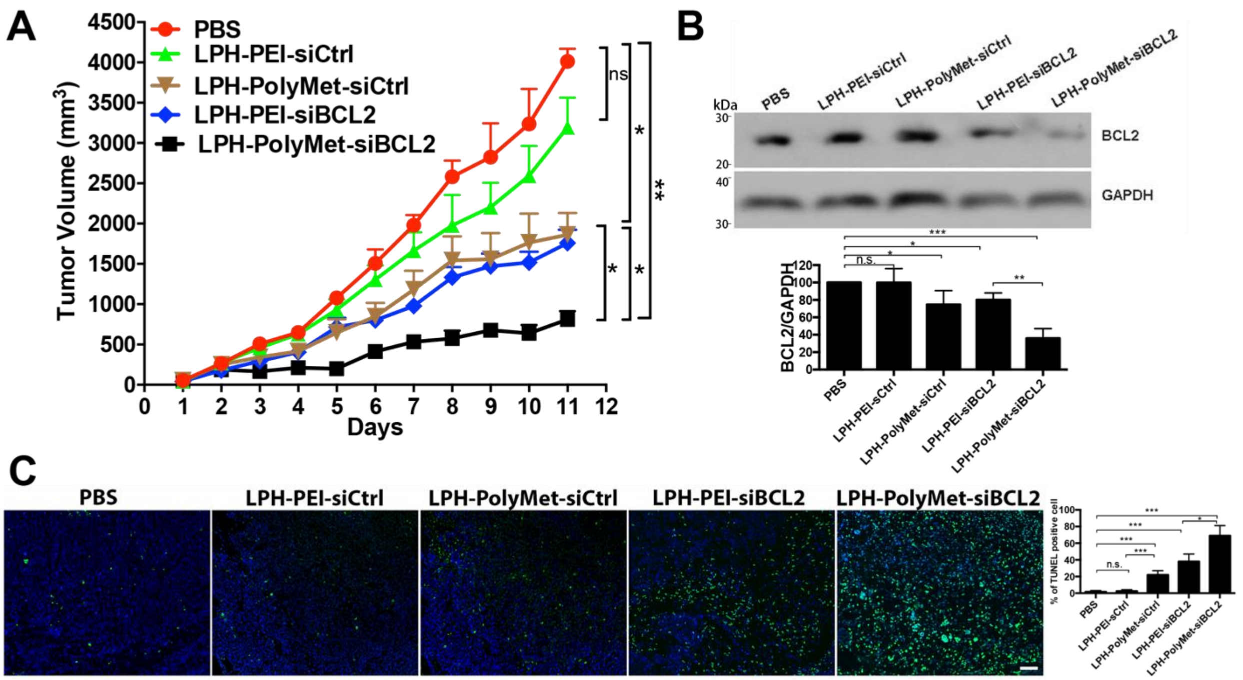

Supplementary Figure 4. *In vivo* gene silencing effect of different LPH nanoparticles. (A) H460 tumor–bearing mice were injected intravenously every other day and tumor volumes were measured every day. (B) H460 tumor BCL2 protein levels after two injections were measured by western blot analysis. Bar chart in B represent quantitative analysis of relative normalized BCL2 band intensity (Image J). (C) TUNEL staining (green) in H460 tumor cells after treatment with siRNA in different formulations *in vivo*. Nucleic acid was stained with DAPI (blue). Bar chart in C is a quantitative analysis of % of TUNEL positive cells. Five randomly selected microscopic fields were quantitatively analyzed on Image J. Data are mean ± SEM (n = 5 per group) analyzed by two-way ANOVA with Tukey's correction. Data are combined from (A) or representative of (B and C) three independent experiments. n.s = not significant, *P < 0.05, **P < 0.01, ***P < 0.005. Scale bar: 200 μm

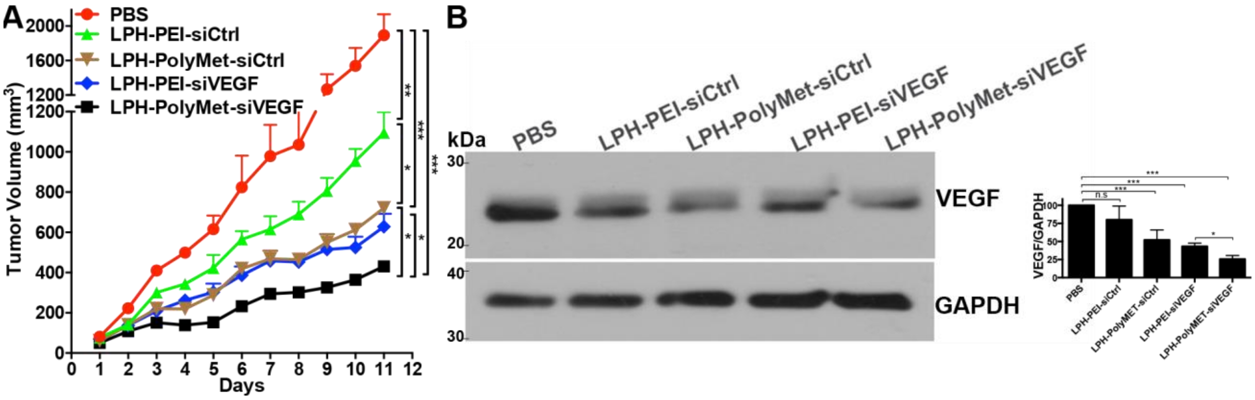

Supplementary Figure 5. *In vivo* gene silencing effect of different LPH nanoparticles in 1205Lu melanoma xenografts. (A) 1205Lu tumor–bearing mice were injected intravenously every other day and tumor volumes were measured every day. (B) 1205Lu tumor VEGF protein levels after two injections were measured by western blot analysis. Bar chart in B represent quantitative analysis of relative normalized VEGF band intensity (Image J). Five randomly selected microscopic fields were quantitatively analyzed on Image J. Data are mean ± SEM (n = 5 per group) analyzed by two-way ANOVA with Tukey's correction. Data are combined from (A) or representative of (B) three independent experiments. n.s = not significant, $^*P < 0.05$, $^{**}P < 0.01$, $^{***}P < 0.005$.

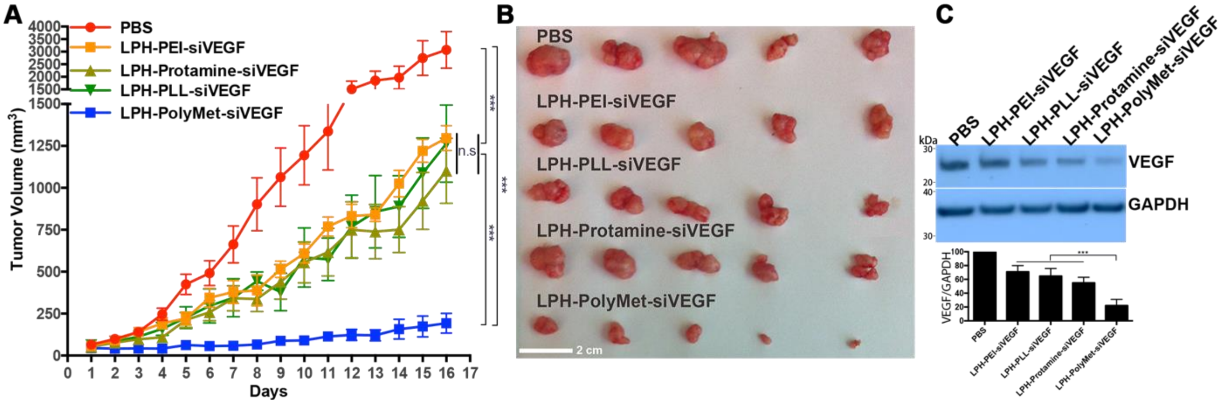

Supplementary Figure 6. *In vivo* gene silencing effect of different LPH nanoparticles in H460 xenografts. (A) H460 tumor–bearing mice were injected intravenously every other day and tumor volumes were measured every day. (B) H460 tumors in each treatment group at the end time point; (C) H460 tumor VEGF protein levels after eight injections were measured by Western blot analysis. Bar chart in C represent quantitative analysis of normalized VEGF band intensity using Image J. Data are mean ± SEM (n = 5 per group) analyzed by two-way ANOVA with Tukey's correction. Data are combined from (A) or representative of (B and C) three independent experiments n.s = not significant, *P < 0.05, **P < 0.01, ***P < 0.005.

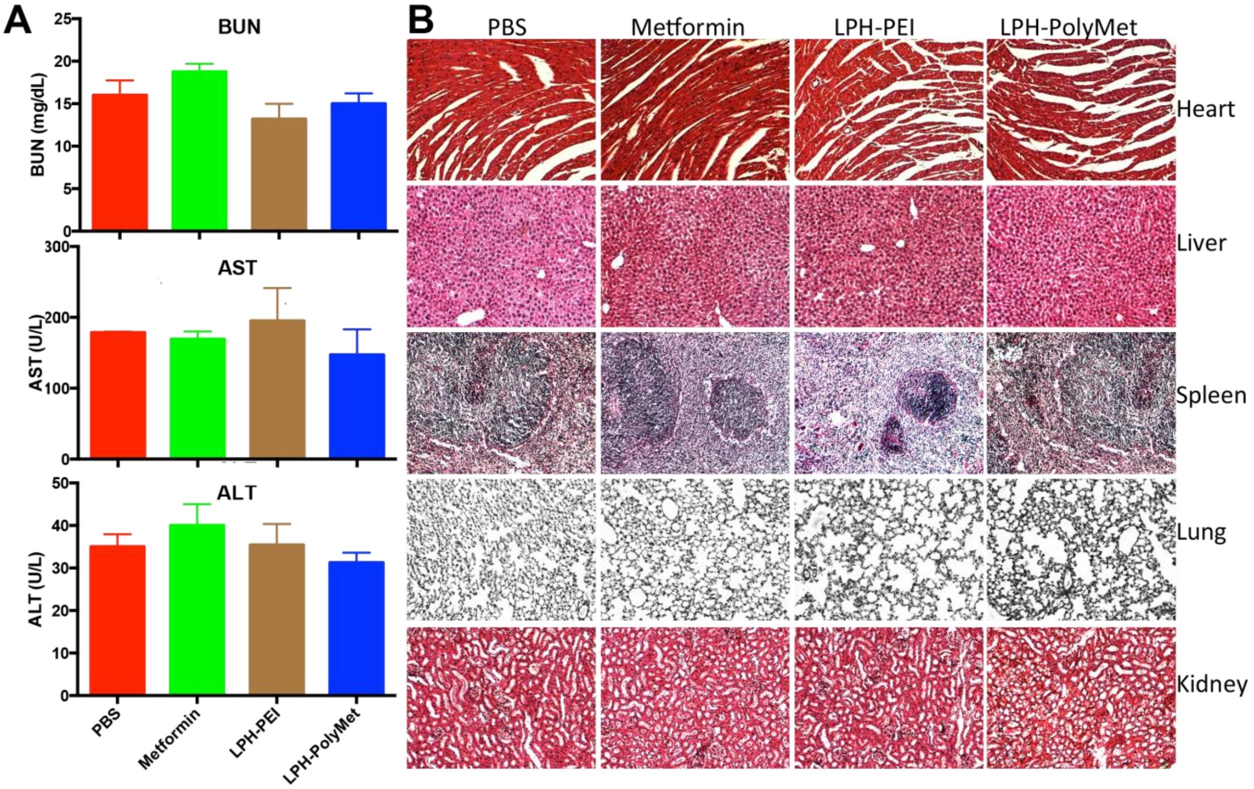

Supplementary Figure 7. (A) Blood biochemistry test of serum and (B) H&E staining of major organs collected from after injections of PBS, Metformin, LPH-PEI and LPH-PolyMet. Data are mean ± SEM (n = 5 per group) analyzed by two-way ANOVA with Tukey's correction. Data are combined from (A) or representative of (B) three independent experiments.

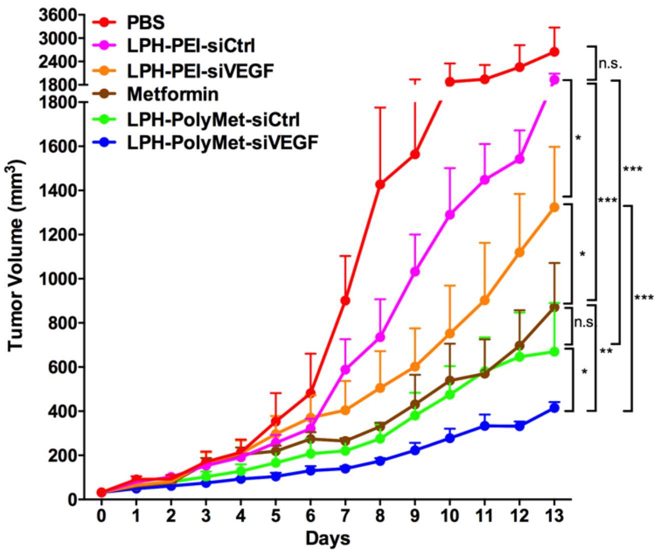

Supplementary Figure 8. H460 tumor–bearing mice were injected intravenously with Metformin and LPH nanoparticles composed of PolyMet or PEI every other day. Tumor volumes were measured every day. Data are mean ± SEM analyzed by two-way ANOVA with Tukey's correction; Data is combined from three independent experiments; 5 mice per group per experiment, n.s. = not significant, *P < 0.05, **P < 0.01, ***P < 0.005.

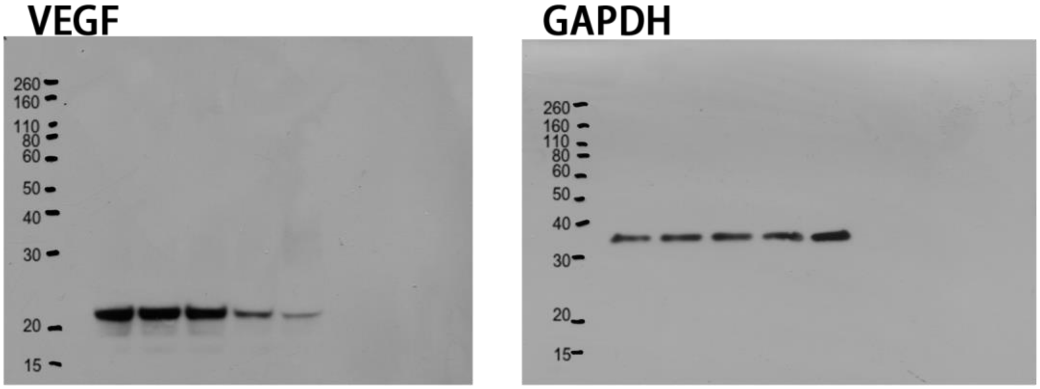

Supplementary Figure 9 *(uncropped scans of Figure 3B)*

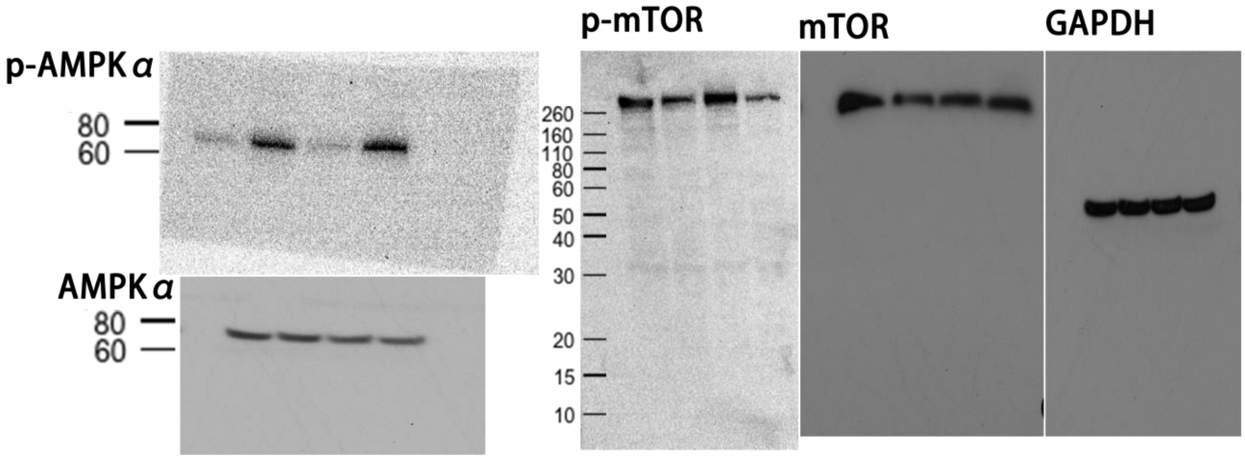

Supplementary Figure 10 *(uncropped scans of Figure 5A)*

Supplementary Tables

Supplementary Table 1. Maximum tolerable dose (MTD) of PEI, PolyMet and Metformin.

Treatments	MTD*, mg kg^{-1}
PEI	2.5
PolyMet	11.5
Metformin	>12

CD-1 mice were IV injected with various concentrations of polymer in 0.9% NaCl solution. At least three animals were tested at each dose. *The MTD is 0.5 mg kg^{-1} below the dose at which any animal died within 24 h of IV injection.

Supplementary Table 2. Dynamic light scattering (DLS) measurement of nanoparticles.

Nanoparticles	Size (nm)	PDI	ζ -potential
PolyMet / (HA+siRNA) complex	103.1 ± 0.4	0.296	-24.1± 0.2
LPH-PolyMet	74.2 ± 0.3	0.215	20.5 ± 0.3

Data is mean ± S.D (n = 3) and represent three independent experiments.

Supplementary Table 3. Hematology test of whole blood collected from after injections of PBS, Metformin, LPH-PEI and LPH-PolyMet.

	PBS	Metformin	LPH-PEI	LPH-PolyMet
WBC	2.2 ± 1.3	2.4 ± 0.8	2.4 ± 0.7	2.4 ± 0.6
Lymphocytes	1.9 ± 0.8	1.5 ± 0.3	1.2 ± 0.6	1.6 ± 0.2
Granulocytes	2.7 ± 0.6	1.4 ± 0.3	1.2 ± 0.3	1.3 ± 0.3
Monocytes	0.6 ± 0.3	0.8 ± 0.3	0.2 ± 0.1	0.4 ± 0.2

Data are mean ± SEM and combined from three independent experiments (n = 5 per group).

Supplementary Methods

Determination of the formation of PolyMet

1) ^{1}H-NMR and ^{13}C-NMR

^{1}H-NMR and ^{13}C-NMR spectra of the PEI-dicyandiamide conjugates and free PEI was performed to determine the synthesis of PolyMet by a NMR instrument (400 MHz). Before analysis, polymers were adjusted to pH 12 with 10 M NaOH, dialyzed against DI-water for 2 days and subsequently lyophilized. Samples were then prepared in anhydrous d6-DMSO solution. The conjugation ratio was determined by comparing the peaks of the conjugate relative to free PEI. ^{1}H-NMR (400 MHz, d6-DMSO): δ 2.59 (s, 2H), δ 2.86-3.75 (br t, 4H), δ 5.94 (br s, 1H), δ 6.07 (br s, 1H), δ 6.19 (br s, 1H).^{13}C-NMR (125 MHz, d6-DMSO): δ 160.9, 160.7, 160.4, 158.8, 158.5, 158.2, 48.1, 47.8, 47.1, 46.8, 45.6.

2) MALDI-TOF

MALDI-TOF of the PolyMet and free PEI was performed to determine the synthesis of PolyMet by using a MALDI-MS instrument (Applied Biosystems 4800). The MALDI-TOF samples were prepared in 50% acetonitrile solution, using a α-Cyano-4-hydroxycinnamic acid matrix. The conjugation ratio was determined by examining the increase in mass of the conjugate relative to free PEI.

Serum Biochemical Value Analysis and Hematology Assay

After three injections, the whole blood was collected and centrifuged at 1,500 g for 5 min to obtain the serum. Blood urea nitrogen (BUN), creatinine, serum aspartate

aminotransferase (AST) and alanine aminotransferase (ALT) levels were assayed as indicators of renal and hepatic function. Whole blood was collected from healthy nude mice after three repeated treatments. Red blood cells (RBC), white blood cells (WBC), platelets (PLT), hemoglobin (HGB) and hematocrits (HCT) were counted for the detection of myelosuppression. Organs (heart, liver, spleen, lung, and kidney) were fixed and sectioned for H&E staining to evaluate organ-specific toxicity.
